# Supplementary material for: The genome sequence of the white-lipped snail, Cepaea hortensis (Müller, 1774) (Stylommatophora: Helicidae)
Source: Wellcome Open Res. 2026 Apr 16;10:681. Originally published 2025 Dec 22. [Version 2] doi: 10.12688/wellcomeopenres.25181.2 (PMC12873540; doi:10.12688/wellcomeopenres.25181.2)
Supplement: Peer review history

# Reviewer report: Reviewer response for version 1

**DOI:** 10.21956/wellcomeopenres.27756.r143435

**Published:** 2026-02-04

**Authors:** Maitrya Sil

**Affiliations:** 1 Jain University, Bengaluru, Karnataka, India

The manuscript is quite well-written. Both the molecular and analytical methods seem quite appropriate for the question. The datasets are also already available online. I don’t have too many comments except that the authors may reassess the completeness using the latest version of BUSCO (version 6). It may help increase the completeness.

Are sufficient details of methods and materials provided to allow replication by others?

Yes

Is the rationale for creating the dataset(s) clearly described?

Yes

Are the datasets clearly presented in a useable and accessible format?

Yes

Are the protocols appropriate and is the work technically sound?

Yes

Reviewer Expertise:

Evolutionary genetics

I confirm that I have read this submission and believe that I have an appropriate level of expertise to confirm that it is of an acceptable scientific standard.

# Reviewer report: Reviewer response for version 1

**DOI:** 10.21956/wellcomeopenres.27756.r143434

**Published:** 2026-01-08

**Authors:** Craig Wilding

**Affiliations:** 1 University College Dublin, Dublin, Ireland

Wade et al. present a high-quality genome assembly for *Cepaea hortensis*, the white-lipped garden snail with this Genome Note providing a useful review of the genome assembly and annotation efforts for this species.

 The background section states that, at least in Ireland and the Pyrenees, the white lip is not a reliable marker for *C. hortensis*. What then is the defining morphological characteristic(s) of this species?

 The methods (Hi-C Sample preparation and crosslinking section) states "Crosslinked DNA was digested with a restriction enzyme master mix". What were the enzymes in this mix?

 The assembly statistic section states "The EBP metric, calculated for the primary assembly, is 6.C.Q53, meeting the recommended reference standard" but the reference BUSCO S metric should be >90 to meet this and for this assembly S=71.4% and C=89.9%. This is not commented on. I note that such low (for a chromosomal assembly) BUSCO score seems typical for Helicids: the BUSCO score for the DToL assembly of *C.nemoralis* is similar - C=88.9% and S=70.4% and that for the DToL assemby of *Cornu aspersum* is C=89.4%  and s = 71.9%. It would be interesting to know whether there is something peculiar about this family, that is why do Helicids seem not to contain something approaching the full BUSCO complement? Or, why does the mollusca_odb10 reference set not seem to be optimal for them?

Are sufficient details of methods and materials provided to allow replication by others?

Yes

Is the rationale for creating the dataset(s) clearly described?

Yes

Are the datasets clearly presented in a useable and accessible format?

Yes

Are the protocols appropriate and is the work technically sound?

Yes

Reviewer Expertise:

Evolutionary genetics

I confirm that I have read this submission and believe that I have an appropriate level of expertise to confirm that it is of an acceptable scientific standard.
